# Impact of Borderline Resectability in Pancreatic Head Cancer on Patient Survival: Biology Matters According to the New International Consensus Criteria

**DOI:** 10.1245/s10434-020-09100-6

**Published:** 2020-09-12

**Authors:** Friedrich Anger, Anna Döring, Jacob van Dam, Johan Friso Lock, Ingo Klein, Max Bittrich, Christoph-Thomas Germer, Armin Wiegering, Volker Kunzmann, Casper van Eijck, Stefan Löb

**Affiliations:** 1 Department of General, Visceral, Transplantation, Vascular and Paediatric Surgery, Julius Maximilians University Wuerzburg, Würzburg, Germany; 2 Department of Surgery, Erasmus MC, University Medical Centre Rotterdam, Rotterdam, The Netherlands; 3 Department of Internal Medicine II, Julius Maximilians University Wuerzburg, Würzburg, Germany; 4 Comprehensive Cancer Centre Mainfranken, Julius Maximilians University Wuerzburg, Würzburg, Germany

## Abstract

**Background:**

International consensus criteria (ICC) have redefined borderline resectability for pancreatic ductal adenocarcinoma (PDAC) according to three dimensions: anatomical (BR-A), biological (BR-B), and conditional (BR-C). The present definition acknowledges that resectability is not just about the anatomic relationship between the tumour and vessels but that biological and conditional dimensions also are important.

**Methods:**

Patients’ tumours were retrospectively defined borderline resectable according to ICC. The study cohort was grouped into either BR-A or BR-B and compared with patients considered primarily resectable (*R*). Differences in postoperative complications, pathological reports, overall (OS), and disease-free survival were assessed.

**Results:**

A total of 345 patients underwent resection for PDAC. By applying ICC in routine preoperative assessment, 30 patients were classified as stage BR-A and 62 patients as stage BR-B. In total, 253 patients were considered *R*. The cohort did not contain BR-C patients. No differences in postoperative complications were detected. Median OS was significantly shorter in BR-A (15 months) and BR-B (12 months) compared with *R* (20 months) patients (BR-A vs. *R*: *p *= 0.09 and BR-B vs. *R*: *p *< 0.001). CA19-9, as the determining factor of BR-B patients, turned out to be an independent prognostic risk factor for OS.

**Conclusions:**

Preoperative staging defining surgical resectability in PDAC according to ICC is crucial for patient survival. Patients with PDAC BR-B should be considered for multimodal neoadjuvant therapy even if considered anatomically resectable.

Ductal adenocarcinoma of the pancreas (PDAC) remains an aggressive gastrointestinal malignancy with a poor prognosis.^1^ Surgical resection, in combination with systemic chemotherapy, offers the only option for long-term survival or even cure for patients with pancreatic cancer. However, modern multimodal treatment approaches still result in 5-year survival rates of 20–30%.^2^ Only 10% of patients diagnosed with PDAC are candidates for upfront resection. Approximately one third of patients present with borderline resectable tumours or locally advanced disease.^3^ Local recurrence rates of 77% attest to the aggressive tumour biology of PDAC. More than 50% recur at single distant sites within the first year after resection suggesting the presence of systemic micrometastases at the time of resection.^4^,^5^

To improve patient selection for surgery in order to achieve optimal R0 resection rates, different guidelines have been developed to define tumour resectability. In 2006, the National Comprehensive Cancer Network (NCCN) introduced criteria that classify PDAC as resectable (*R*), borderline resectable (BR), or unresectable (UR)—the latter includes locally advanced disease (LA) or metastatic disease. With special attention to BR-PDAC, several versions have been adopted over the years. They all share the main definition of BR adhering to the concept of technical resectability with high risk of positive surgical margins and consequently local progression.^6^^–^8 Accumulating evidence indicates that patients with BR-PDAC benefit from neoadjuvant multimodal therapy, including chemotherapy and/or radiation therapy as treatment failure of surgery-first approaches in these patients has been shown to be common in the BR-PDAC patient cohort.^9^,^10^ Consequently, these concepts have been incorporated into the NCCN and further into the European Society for Medical Oncology (ESMO) guidelines.^11^^–^13

Resectability of PDAC was traditionally determined by the surgeon during the trial dissection. Improvements in radiologic imaging have allowed a more accurate preoperative assessment of resectability.^14^ However, both surgical and radiologic criteria of resectability are still based on anatomic criteria alone. Biological and conditional criteria were first described as important players of extended resectability criteria for BR-PDAC in 2008. However, they were not included in treatment guidelines for BR-PDAC patients. In 2014, the International Study Group of Pancreatic Surgery expanded the definition of BR-PDAC by the inclusion of carbohydrate-antigen 19-9 (CA19-9) serum levels as a preoperative biological marker, but not as a criterion for resectability.^15^ Finally, in 2017 international consensus was achieved for the definition of BR-PDAC. According to these criteria, borderline-resectability is defined by each of the following three dimensions: anatomical (A), biological (B), and conditional (C). Biological evaluation is performed through measuring CA19-9 serum levels or prediction of regional lymph node metastasis by cross-sectional imaging. The patient’s condition is evaluated using the eastern cooperative oncology group (ECOG) performance status.^16^ Consequently, patients are considered BR by one dimension or a combination of two or three criteria.^16^ Meanwhile, ICC was validated in two Asian retrospective cohort studies. They proved to be useful and practicable in the determination of borderline resectability in BR-PDAC.^17^,^18^ To date, neither European nor American guidelines have incorporated these criteria.

The purpose of this study was to evaluate retrospectively the impact of the novel consensus criteria defining BR-PDAC compared with current NCCN guidelines on patient survival after upfront pancreaticoduodenectomy (PD).

## Materials and Methods

### Patient Population

All patients that received upfront pancreaticoduodenectomy (PD) for PDAC of the pancreatic head at the University Hospital Wuerzburg, Germany (UKW), and the Erasmus Medical Centre Rotterdam, Netherlands, between 2003 and 2017 were identified from Institutional Database of each hospital. During the study period, selection criteria for upfront surgery where the same in both centres and were based on the NCCN criteria. Patients with distant metastasis, neoadjuvant treatment and/or arterial resection were excluded from the study. Resected specimens were histologically confirmed as invasive ductal adenocarcinoma.

Patients were retrospectively assigned to four groups (BR-A, BR-B, BR-C, or *R*) according to ICC on the classification of BR-PDAC.^16^ This was done stepwise and prioritized according to anatomical criteria, i.e., patients with involvement of venous or arterial vessels were assigned to group BR-A, regardless of their CA19-9 levels. No combinations of different ICC dimensions were calculated (e.g., BR-AB). In detail, patients with a tumour contact of less than 180° to the superior mesenteric artery (SMA) or common hepatic artery (CHA) and/or a tumour contact of 180° or greater to the superior mesenteric vein (SMV) or portal vein (PV), bilateral venous narrowing, venous occlusion, or tumour growth exceeding the inferior border of the duodenum on the line of SMV in preoperative cross-sectional imaging were exclusively assigned to the BR-A group. Patients with preoperative CA19-9 serum levels that exceeded 500 U/ml and who had not been assigned to the BR-A group were allocated to the BR-B group. Preoperative positron emission and computed tomography (PET-CT) or endoscopic ultrasound to rule out potential regional lymph node metastasis had not been performed on a regular basis and were therefore not accounted for in the definition of BR-B patients. No patients with an Eastern Cooperative Oncology Group (ECOG) status of ≥ 2 underwent PD for PDAC at both hospitals. Consequently, no BR-C group was to be formed. All other patients’ tumours were staged primarily resectable (maximum vein abutment < 180°) and assigned to group *R*. The primary endpoint of this study was the difference in overall-(OS) and disease-free survival (DFS) between groups. Secondary endpoints were type of surgery, the incidence of postoperative complications according to the Clavien-Dindo-Score,^19^ length of hospital stay, and 30-day mortality. Additionally, the rate of preoperative bile drainage and differences in pathological reports were assessed and the numbers of patients who had undergone adjuvant chemotherapy were recorded.

### Data Source

The central prospective databases of both centres provide data on patient demographics, histological diagnoses based on International Classification of Diseases coding standards, physician data, inpatient admission and outpatient registration data, operative procedures, laboratory values, and computerized medication records. Updated follow-up information is ensured by continuous cross platform integration with the Wuerzburg Comprehensive Cancer Registry and the Netherlands Cancer Registry for the identification of deceased patients. The records of all patients identified were reviewed retrospectively regarding adjuvant chemotherapy, sites of metastatic disease at presentation, disease status at last follow-up, or any missing data. Patients’ demographic details and clinical variables recorded at the time of primary diagnosis as well as during the initial operation (tumour site and the presence of any metastases) were compiled. Histological details of resected specimens were categorized according to the TNM staging system of pancreatic cancer (8th edition) of the American Joint Committee on Cancer/Union Internationale Contre le Cancer (AJCC/UICC-TNM) as follows: tumour (T) stage, nodal (N) stage, tumour differentiation (G), and evidence of microscopic venous (V), lymphatic vessel (L), or perineural invasion (Pn)). The reporting pathology system on the resection status has changed over the study period. Therefore, R0 resection status was defined as no detectable tumour cells at the transection or circumferential margin (CRM) according to the currently valid definition of “CRM narrow.”

### Treatment

All patients underwent physical examination and were staged by multidetector computed tomography of the thorax and abdomen to rule out distant metastases. Patients with total bilirubin levels > mg/dl underwent biliary drainage prior to surgery. In case of limited obstructive jaundice (< 15 mg/dl) and missing signs of acute cholangitis primary resection was performed in patients with suspected PDAC. For this study, we used only CA19-9 levels after stent placement and as close as possible before the day of surgery. All patients underwent pancreaticoduodenectomy (PD) that was either pylorus-resecting according to Kausch and Whipple or pylorus-preserving according to Traverso and Longmire as well as systematic lymphadenectomy. In some patients, complete pancreatectomy was necessary to achieve tumour-free resection margins. All patients were discussed in a multidisciplinary team conference at the time of diagnosis and after surgery. Adjuvant treatment consisted of Gemcitabine 1000 mg/m^2^, 6 cycles of 3 weeks followed by 1-week rest.^20^

### Follow-Up

The German national (AWMF) and European (ESMO) guidelines do not recommend a regular follow-up after initial therapy with curative intent.^11^,^12^ Nevertheless, most patients treated at both centres underwent reevaluation of postoperative tumour burden by cross section imaging before or after adjuvant chemotherapy. Postoperative follow-up consisted of outpatient assessments on demand or the gathering of complete information from patients’ primary care physicians. CT scans were performed on demand whenever recurrence was suspected based on the patients’ physical condition, complaints, or elevated tumour markers. OS was defined as the time period from the date of surgery to the date of death by any cause. DFS was defined as a time period from the date of surgery to the date of tumour recurrence or death. Patients who died postoperatively were excluded from the survival analysis.

### Statistical Analysis

Data were analysed with IBM SPSS Statistics Version 25. Clinical and histological parameters were compared with the analysis of variance (ANOVA), Chi square, and Fisher exact test, as appropriate. Survival curves were drawn according to Kaplan–Meier methods. Log-rank test was used for comparison of survival analysis. Holm-Bonferroni correction was applied to accommodate multiple testing in subgroup analysis.^21^,^22^ Preoperative and pathological variables with a *p* value < 0.1 in the univariate analysis were included into a Cox proportional hazards model using a backward stepwise selection to determine the independent risk factors associated with OS and DFS. A *p* value< 0.05 was considered statistically significant.

## Results

### Patient Characteristics

Between January 2003 and December 2017, 345 patients were diagnosed with adenocarcinoma of the pancreatic head and met the inclusion criteria. The cohort consisted of 208 male and 137 female patients, with a median age of 69 (range 33–90) years and a median preoperative body mass index (BMI) of 25.0 kg/m^2^ (range 16–41). The most common ASA scores were 2 and 3 (89.6%). Most patients presented with obstructive jaundice (72.3%). Consequently, serum bilirubin-levels were raised to 1.8 mg/dl in the median ranging from 0.2 to 40.4 mg/dl. A total of 197 patients (57.1%) underwent preoperative bile drainage. Patients received biliary stenting either during the initial diagnostic workup in a community hospital or because of pending cholangiosepsis. The median serum CA19-9 level was 132 U/l (range 0.6-23898). According to the ICC, 92 patients were staged BR: 30 patients (8.7%) due to anatomical criteria (BR-A) and 62 patients (18.0%) due to CA19-9 serum levels exceeding 500 U/l (BR-B). Patient characteristics are listed in Table 1.

**Table 1:** Patient characteristics

Patient population	All	BR-A	BR-B	*R*	*p* value
No. of patients (*n*, %)	345	30 (8.7)	62 (18.0)	253 (73.3)	
Age (year, median (min–max))	69 (33–90)	66 (35–88)	70 (49–82)	69 (33–90)	0.181
Gender male (*n*, %)	208 (60.3)	15 (50.0)	39 (62.9)	154 (60.9)	0.463
BMI (kg/m^2^, median (min–max))	25.0 (16.1–41.1)	24.0 (19.6–30.9)	25.1 (16.5–39.3)	25.1 (16.1–41.1)	0.471
ASA score (*n*, %)					
1	18 (5.2)	3 (10.0)	2 (3.2)	13 (5.1)	
2	190 (55.1)	21 (70.0)	31 (50.0)	143 (56.5)	
3	119 (34.5)	6 (20.0)	27 (43.5)	86 (40.0)	
4	3 (0.9)	0 (0)	2 (3.2)	1 (0.4)	0.107
Bilirubin (mg/dl, median (min–max))	1.8 (0.2–40.4)	1.8 (0.5–18.2)	7.1 (0.4–29.2)	1.4 (0.2–40.4)	**0.001**
Preoperative biliary stent (*n*, %)	197 (57.1)	17 (56.7)	27 (43.5)	153 (60.5)	0.054
CA19-9 (U/l, median (min–max))	132 (1–23898)	145 (1–7408)	997 (502–23898)	76 (0.6–500.0)	**<** **0.001**
Median overall survival (mo, median (95% CI))	18 (15.9–20.1)	15 (8.0–22.0)	12 (8.9–15.0)	20 (17.6–22.4)	**<** **0.001**
Median disease-free survival (mo, median (95% CI))	11 (9.2–12.8)	5 (1.6–8.4)	8 (6.4–9.6)	13 (11.4–14.6)	**<** **0.001**

### Surgery and Postoperative Complication Rate

In both departments, pylorus-preserving PD is the standard procedure for pancreatic head resections. Technical variations in terms of additional distal gastrectomy or completing-pancreatectomy are based on the surgeon’s intraoperative decision and/or frozen-section results to achieve tumour-free resection margins. Consequently, 203 (58.8%) pylorus-preserving PD, 123 (35.6%) pylorus-resecting PD and 19 (5.5%) total pancreatectomy procedures were performed. In the BR-A group, complete pancreatectomy as a consequence of repeated positive resection margins on frozen sections was performed significantly more often (16.6%) compared with the BR-B (8.0%) and *R* (3.5%, *p *< 0.010) groups. Overall postoperative morbidity was 53.6%. We did not detect intergroup variations with regard to total complications. However, patients in the BR-A group developed significantly less major postoperative complications (Clavien-Dindo ≥ Grade IIIa).

There was no difference in length of hospital stay between groups. The 30-day mortality rate was 0% in the BR-A group (0%), 8.1% in the BR-B (8.1%), and 2.8% in the *R* group (*p *= 0.069). Table 2 summarizes the information on postoperative morbidity and mortality rates between groups.

**Table 2:** Perioperative and pathological parameters according to patient subgroups BR-A, BR-B, and *R*

	BR-A *n* = 30	BR-B *n* = 62	*R* *n* = 253	*p* value
*Surgery and postoperative complications*				
Traverso–Longmire (*n*, %)	11 (36.7)	37 (59.7)	155 (61.3)	
Kausch–Whipple (*n*, %)	14 (46.7)	20 (32.3)	89 (35.2)	
Pancreatectomy (*n*, %)	5 (16.6)	5 (8.0)	9 (3.5)	**0.010**
Total operation time (min, median (min–max))	379 (237–634)	363 (206–589)	351 (195–756)	0.099
Clavien-Dindo ≥ IIIa (*n*, %)	2 (6.7)	17 (27.4)	87 (34.4)	**0.007**
Reoperation rate (*n*, %)	2 (6.7)	7 (11.3)	37 (14.6)	0.418
Length of hospital stay (days, median (min–max))	12.5 (3–37)	19 (7–61)	18 (5–173)	**0.017**
30-day mortality	0 (0.0)	5 (8.1)	7 (2.8)	0.069
*Pathological reports*				
Tumour status (*n*, %)				
T1	3 (10.0)	9 (14.5)	47 (18.6)	
T2	18 (60.0)	43 (69.4)	166 (65.6)	
T3	9 (30.0)	10 (16.1)	40 (15.8)	0.296
Largest tumor diameter (cm, median (min–max))	3.6 (1.5–7.5)	3.0 (1.0–6.0)	2.8 (0.2–8.0)	**0.003**
Lymph node status (*n*, %)				
N0	11 (36.7)	7 (11.3)	55 (21.7)	
N1	11 (36.7)	37 (59.7)	154 (60.9)	
N2	8 (26.6)	18 (29.0)	43 (17.0)	**0.010**
Lymphovascular tumour invasion (*n*, %)				
L0	5 (16.7)	20 (32.3)	73 (28.9)	
L1	25 (83.3)	42 (67.7)	180 (71.2)	0.681
Perineural tumour invasion (*n*, %)				
Pn0	1 (3.3)	13 (21.0)	44 (17.4)	
Pn1	29 (96.7)	49 (79.0)	209 (82.6)	0.140
Resection status (*n*, %)				
R0	16 (53.3)	37 (54.7)	203 (80.2)	
R1	14 (46.7)	25 (40.3)	50 (19.8)	**0.001**
Tumour grading (*n*, %)				
G1	3 (10.0)	2 (3.2)	19 (7.5)	
G2	14 (46.7)	29 (46.8)	165 (65.2)	
G3	13 (43.3)	28 (45.2)	68 (26.8)	
G4	0 (0.0)	3 (4.8)	1 (0.4)	**0.003**
UICC stage (*n*, %)				
1a	3 (10.0)	3 (4.8)	19 (7.5)	
1b	5 (26.7)	4 (6.5)	31 (12.3)	
2a	3 (10.0)	0 (0.0)	8 (3.2)	
2b	11 (36.7)	37 (59.7)	153 (60.5)	
3	8 (26.6)	18 (29.0)	42 (16.6)	0.074
Adjuvant chemotherapy (*n*, %)	15 (50.0)	27 (43.5)	125 (49.4)	0.634

### Pathological Reports and Adjuvant Chemotherapy

According to the tumour-node-metastasis system (8th edition of TNM^23^) introduced by the AJCC/UICC-TNM, most patients were classified into T2 (65.8%) and N1 (58.5%) categories. No differences in UICC stages were observed between groups. Median tumour diameter was significantly larger in the BR-A group (*p *= 0.003), but tumour stages (T) did not differ significantly between groups (*p *= 0.296). BR-A (63.3%) and BR-B (88.7%) patients were significantly more often nodal-positive than patients in the *R* group (57.1%) (*p *= 0.010). The highest N2 rates were detected among BR-B patients (29.0%). There were marked intergroup differences in positive resections margins and tumour grading (G). Pathological examination of pancreatic head specimens revealed that positive resection margins (R1) occurred significantly more often in patients staged BR-A (46.7%) and BR-B (40.3%) than in those tumours staged primarily resectable (19.8%, *p *= 0.001). Patients classified into BR-A or BR-B showed significantly worse tumour grading (G3/G4) than those assigned to the *R* group (*p *= 0.003). No difference with regard to perineural or lymphatic vessel infiltration rates was detected. Approximately 50% of patients received adjuvant chemotherapy in either group. Detailed pathologic information is outlined in Table 2.

### Oncological Outcome and Risk Factors of Overall Survival

**Fig. 1:**
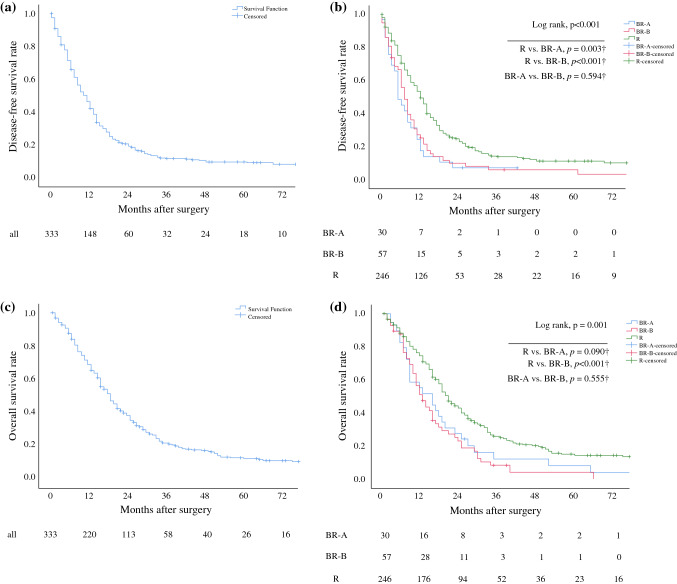
**a**, **c** Disease-free and overall survival of all patients included. **b**, **d** Disease-free and overall survival by preoperative staging according to ICC. Survival curves are drawn according to Kaplan–Meier with patients at risk below each graph. *p* < 0.5 was defined statistically significant. ^†^In terms of multiple comparisons for subgroup analysis (BR-A vs. *R*, BR-B vs. *R*, BR-A vs. BR-B), each *p*-value has been corrected according to the Holm–Bonferroni method

Lost to follow-up was 10.0% in the BR-A group, 12.3% in the BR-B group, and 19.9% of patients in the *R* group. With regard to the entire study population, the median disease-free survival (DFS) was 10 months (Fig. 1a) and overall survival (OS) was 18 months (Fig. 1c). The median DFS was significantly shorter in patients staged BR-A (5 months) and BR-B (7 months) compared with patients staged *R* (12 months, BR-A vs. *R*: *p *= 0.003, BR-B vs. *R*: *p *< 0.001; Fig. 1b). The median OS was significantly shorter in patients staged BR-B (12 months) and showed a clear trend towards a shorter OS in the BR-A group (15 months) compared with patients staged *R* (20 months, BR-B vs. *R*: *p *< 0.001, BR-A vs. *R* = 0.09, Fig. 1d).

**Fig. 2:**
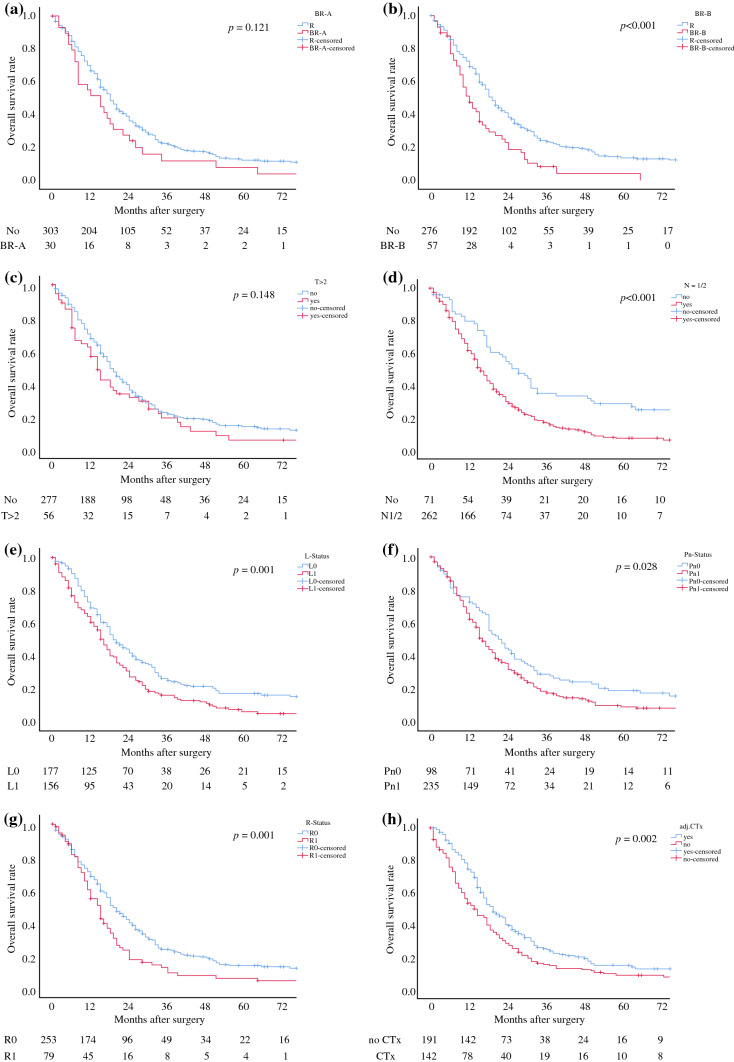
Overall survival by **a** BR-A, **b** BR-B, **c** T-status > 2, **d** lymph node metastasis, **e** lymphatic vessel infiltration, **f** perineural infiltration, **g** resection status, and **h** adjuvant chemotherapy (CTx) of all patients included into survival analysis. Survival curves are drawn according to Kaplan–Meier with patients at risk below each graph

**Fig. 3:**
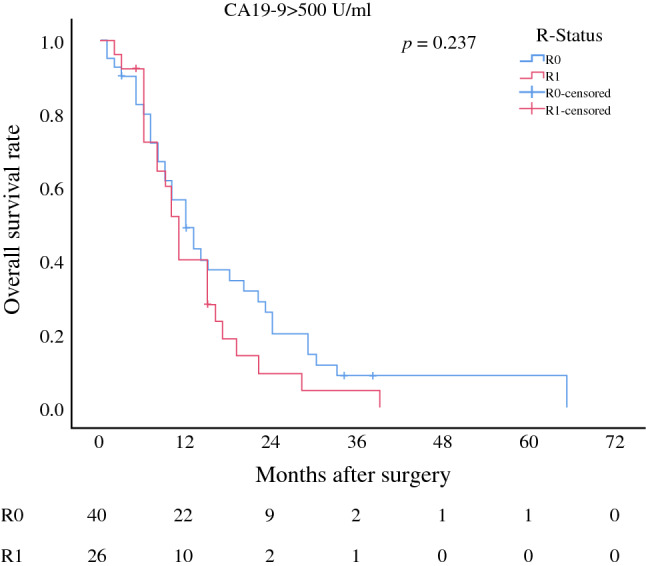
Overall survival by resection status (R0 vs. R1) in patients with preoperative CA19-9 serum-level above 500 U/ml. Survival curves are drawn according to Kaplan–Meier with patients at risk below each graph

First, the two dimensions of BR and several histological factors were analysed for their prognostic impact on OS in univariate analysis (Fig. 2; Table 3a). BR-B (*p *< 0.001), a nodal-positive PDAC (*p *< 0.001), a positive lymphovascular (*p *= 0.001) or perineural infiltration (*p *= 0.028), positive resections margins (*p *= 0.001), or no adjuvant chemotherapy (*p *= 0.002) were associated with a significantly worse overall survival of PDAC patients. Interestingly, subgroup analysis of all patients with preoperative CA19-9 serum values above 500 U/ml showed that the resections margins (R0 vs. R1) lost impact on overall survival in this patient cohort (Fig. 3). In multivariable analysis, BR-B (HR 1.53, *p *< 0.001) together with lymph node metastasis (HR 1.92, *p *< 0.001), lymphovascular invasion (HR 1.68, *p* = 0.001), positive resection margins (HR 1.43, *p *= 0.012), and failure to adjuvant chemotherapy (HR 1.73, *p *< 0.001) turned out to be independent prognostic risk factors of OS (Table 3a).

**Table 3:** Univariate and multivariable analysis

Clinical factor	Patients *n* = 333	Univariate *p*	Multivariable		
			HR	95% CI	*p*
*(a) Pre*- *and postoperative parameters associated with OS*					
BR-A	30	0.121			
BR-B	57	**<** **0.001**	1.53	1.11–2.11	0.010
T-Status > 2	56	0.148			
N-Status ≥ 1	262	**<** **0.001**	1.94	1.42–2.67	**<** **0.001**
L-Status = 1	156	**0.001**	1.38	1.08–1.78	**0.012**
Pn-Status = 1	235	**0.028**	1.16	0.87–1.53	0.313
*R*-Status = 1	79	**0.001**	1.43	1.08–1.89	**0.012**
no adj. CTx	142	**0.002**	1.73	1.35–2.21	**<** **0.001**
*(b) Pre*- *and postoperative parameters associated with DFS*					
BR-A	30	**0.005**	2.22	1.47–3.34	**<0.001**
BR-B	57	**0.001**	1.42	1.05–1.94	**0.025**
T-Status > 2	56	0.117			
N-Status ≥ 1	262	**<** **0.001**	1.85	1.36–2.52	**<** **0.001**
L-Status = 1	156	**<** **0.001**	1.58	1.24–2.02	**<** **0.001**
Pn-Status = 1	161	0.064	1.05	0.80–1.39	0.693
*R*-Status = 1	49	**0.023**	1.11	0.84–1.47	0.464
no adj. CTx	83	**0.012**	1.64	1.28–2.09	**<** **0.001**

Second, BR-A (*p *= 0.005), BR-B (*p *= 0.001), N1/2 (*p *< 0.001), L1 (*p *< 0.001), Pn1 (*p *= 0.064), R1 (*p *= 0.023), and no adjuvant chemotherapy (*p *= 0.012) were identified as prognostic risk factors for DFS in univariate analysis (Table 3b). A multivariable analysis revealed lymph node metastasis (HR 1.85, *p *< 0.001), lymphovascular invasion (HR 1.58, *p *< 0.001), no adjuvant chemotherapy (HR 1.64, *p *< 0.001), BR-A (HR 2.22, *p *< 0.001), and BR-B (HR 1.4, *p *= 0.025) as independent prognostic risk factors for DFS (Table 3b).

## Discussion

There is accumulating evidence that neoadjuvant treatment protocols result in a survival benefit for patients with BR-PDAC.^9^,^24^ However, most studies were nonrandomized, BR-definitions heterogenous and only attributed to anatomical borderline-resectability. So far, three RCTs have been published that have analysed the prognostic impact of different neoadjuvant treatment protocols in anatomically borderline-resectable and primarily resectable PDAC patients.^25^^–^27 Results of the Korean and Japanese trials showed a survival benefit especially for BR PDAC patients,^25^,^26^ whereas the European trial did not show a significantly improved overall survival. However, survival analysis of patients who underwent tumour resection and started adjuvant chemotherapy showed improved survival with preoperative chemoradiotherapy.^27^ In this study, we retrospectively assigned patients from two European centres with BR PDAC to different preoperative groups according to the new International consensus criteria (ICC). Although the association of CA 19-9 elevation and outcome has been demonstrated in numerous series,^28^ we were able to show for the first time in a European patient cohort that biology based on preoperative CA 19-9 levels is at least as important as anatomy in defining resectability of patients with adenocarcinoma of the pancreatic head. This study demonstrated that preoperative staging of borderline resectability according to the new international consensus criteria resulted in significant survival differences for subsets of patients who had undergone upfront resection surgery for PDAC. First, tumour involvement along the portal and/or mesenteric vein dramatically reduced DFS and OS, which was comparable to studies that included patients with BR-PDAC and venous or arterial invasion.^9^,^18^ Second, we identified a subset of patients that was classified BR from having elevated CA 19-9 serum levels with an equal or even stronger reduction in DFS and OS as patients with anatomically borderline resectable disease. However, according to current guidelines, such as NCCN or ESMO, these patients would still be scheduled for upfront resection. In contrast, our results clearly indicate that disease-free and overall survival of BR-B patients is at least as compromised as of patients who were classified BR due to anatomic reasons (BR-A). According to our results, ICC turned out to be more precise in defining resectability in PDAC patients according to their survival analysis. Therefore, ICC should be implemented in diagnostic and treatment algorithms for patients with pancreatic head cancer.

In this context, some aspects of the ICC need to be addressed. Regarding anatomical criteria to define resectability of PDAC, NCCN, and ESMO guidelines are both based on tumour involvement of vessels along the porto-mesenteric axis.^12^,^13^ ICC extend these criteria by a further anatomic landmark in preoperative imaging, i.e., the inferior border of the duodenum on the line of SMV.^16^ Tumour growth beyond this landmark would account for locally advanced disease and no curative resection option. It must be stated that this consensus is rather weak, because it was based on only two patients who did not undergo resection for that reason.^16^ In our study, none of the patients staged BR-A presented with tumour growth exceeding the inferior border of the duodenum. As expected, BR-A patients showed a significantly higher number of nonradical resections (R1). Interestingly, this also was true for BR-B patients who had been preoperatively staged anatomically resectable. Probably the preoperative imaging might have underestimated the extent of the tumour size in patients with high preoperative CA19-9 levels. However, our data confirm previous reports that preoperatively elevated CA19-9 levels correlate with the resection status of the pancreatic head specimen with R1 resections rate of 55% for patients with CA19-9 above 500 U/ml.^29^ Consequently, our results support current NCCN guidelines recommending neoadjuvant therapy at least for BR-A patients, as this therapeutic algorithm significantly reduces R1 resection rates in BR-A patients.^25^,^30^ Because the definition of R0 has changed over time and the concept of a circumferential resection margin (CRM) has been introduced during the study period, we defined R0 resection status as no detectable tumour cells at the transection or circumferential margin (CRM) according to the currently valid definition of “CRM narrow.” This might have resulted in an overall underrepresentation of positive resection margins and a possible bias in survival data, as the redefinition of a negative resection margin by the German guideline in 2013 led to a significant reduction of so called curative resections in large patient cohorts.^31^ The higher rate of Clavien-Dindo ≥ IIIa complications in the *R* and BR-B group compared with BR-A might be due to the consistency of the pancreatic remnant and duct size, which is usually soft in patients with anatomically resectable tumor without pancreatic duct obstruction. Some studies reported about the prognostic impact of postoperative complications following pancreatic head resections on overall survival with inconsistent results.^32^,^33^ Nonuniform grading systems for postoperative complications and study populations, including patients after neoadjuvant treatment who per se show an impaired survival compared with primarily resectable PDAC patients, might have influenced different outcome results. Treatment delays or even omission of adjuvant chemotherapies have been discussed to influence overall survival.^34^ Although complication rates were different between groups in this study, approximately 50% of patients received adjuvant chemotherapy in all groups.

The main novelty in defining BR-PDAC according to ICC constitutes the incorporation of preoperative CA19-9 serum levels as a function of tumour biology. By applying the proposed cutoff value of 500 U/ml, we identified 62 patients in our cohort. Interestingly, these patients presented with a larger extend of lymph node metastasis, a higher R1 resection rate, worse tumour grading, and shortened DFS and OS compared with patients staged *R* according to ICC. These findings indeed indicate to a more aggressive tumour biology of BR-B PDAC. In a retrospective analysis of Japanese patients who had also received upfront resection surgery for PDAC, Kato et al. proposed a CA19-9 cutoff value of 1000 U/l for the definition of borderline resectability according to the multivariate analysis.^18^ In our study, CA19-9 values were set as suggested by ICC and proved to be an independent prognostic marker for DFS and OS. Most interestingly, the impact of the resection status on overall survival gets lost in this patient cohort. Patients with serum CA19-9 levels of more than 500 U/ml do not show differences in overall survival according to the resection status (R0 vs. R1). Of note, the ICC consensus statement is based on a large German cohort analysis, describing a continuous decline in resection rate and median survival time with a rise in preoperative CA19-9 serum levels.^29^ It is known that the positive predictive value of CA19-9 in order to determine malignant disease is 72.3%^35^ and that 7-10% of the Caucasian population are non-secretors due to their ABO-type.^36^ This might result in an underrepresentation of biologically aggressive tumours. But CA19-9 has been used in risk profiling of PDAC patients for years and—to our knowledge—there is no alternative serum marker in PDAC patients available so far that resembles tumour biology more reliably than CA 19-9. Another advantage of CA 19-9 is that it allows a first approximation of the individual tumour biology without the need for a tumour biopsy, because it serves as a prognostic marker in different malignant entities of the pancreatic head (e.g., distal cholangiocarcinoma^37^). We do respect that a large variety of genetic and molecular variations have been identified in pancreatic cancer (i.e., K-ras, p16, p53, BRCA2, smad4 genes) in the past.^38^ However, translation of this scientific knowledge into clinical treatment regimen is still largely unrealized. At present, serum CA19-9 may therefore qualify best. There also have been conflicting reports on the interaction of CA19-9 and hyperbilirubinemia as increased pressure on the common bile duct has been postulated to be linked with increased CA19-9. Relief of jaundice was described to be associated with a decrease of CA19-9 in a substantial amount of patient with benign and malignant diseases.^39^,^40^ Consequently, subsequent studies have ruled out patients with increased bilirubin levels or have calculated adjusted CA19-9 levels.^41^ A more recent study on a large patient PDAC patient population confirmed this correlation, but more importantly found that the correlation coefficient was very low.^29^ The authors concluded that adjusted CA19-9 levels are not mandatory. Appropriate cutoff values might vary within different patient cohorts and therefore need to be further analysed in prospective studies. Furthermore, because all patients with bilirubin levels > 15 mg/dl underwent biliary drainage before surgery in this study, we used CA19-9 levels determined just before surgery when bilirubin levels were normalised in most patients.

Apart from CA19-9 levels, ICC considers preoperative lymph node evaluation in terms of metastases for the definition of biological borderline resectability in PDAC patients (BR-B). In our study, the preoperative evaluation for the likelihood of lymph node metastases was not assessed by PET-CT and lymph node biopsy was not routinely performed. Lymph node metastasis is a well-known independent prognostic factor^42^,^43^—as underlined by our results. However, reliable prediction of lymph node metastasis in PDAC remains challenging.^44^ The highest negative predictive value of 93.3% for preoperative determination of lymph node metastasis in pancreatobiliary tumours was reported from MRI studies.^45^ These promising results should encourage further improvements in preoperative imaging techniques with the chance to classify patients with greater accuracy into BR-B according to lymph node status. In our patient cohort, N2-category occurred twice as often in patients staged BR-B compared with other groups. Possibly, CA19-9 serum levels might additionally help to assess the extent of lymph node metastases preoperatively.^46^

The following issues need to be addressed. First, because this is a retrospective study and lost to patient follow-up was considerable, patient selection bias cannot be ruled out, especially because the approach to patients with vascular involvement changed over time to neoadjuvant therapy. Because upfront resections in patients with borderline disease has only been recommended since the 2016 version of the NCCN guidelines, most patients with BR-A disease underwent an explorative laparotomy. However, the results of this study confirm those reported by Kato et al.,^18^ who showed an impaired survival of patients staged BR-B compared with patients staged *R* according to ICC by using a similar study design. Nevertheless, prospective validation of the current results along with calculation of appropriate cutoff values for CA19-9 serum levels is needed together with describing the recurrence pattern in both groups to define the poor prognosis of the BR-B group. Second, due to low patient numbers (*n* = 6) we did not analyse BR-AB patients as an independent group but included them in the BR-A group. In these patients, the impact of tumour biology in terms of elevated CA19-9 levels might have been trumped. Third, due to the overall low number of patients who received adjuvant treatment as a reflection of changes in national recommendations for the treatment of PDAC over the study period, we observed a rather short median DFS and OS. Consequently, missing adjuvant treatment was an independent prognostic risk factor for DFS and OS. However, distribution of patients who received adjuvant treatment was equal between groups and thus does not explain the differences in OS. Fourth, the preoperative staging of PDAC improved over time thereby increasingly determining treatment proposals in multidisciplinary conferences, ultimately evolving the concept of multimodal neoadjuvant therapy for borderline resectable PDAC with considerable oncological benefit.^25^ Given the fact that evaluation of patients with PDAC varies between different interdisciplinary conferences,^47^ resection criteria for PDAC should be defined more precisely. Because the outcome of the so-called biological borderline group (preoperative CA 19-9 serum levels > 500 U/ml) is so poor, new neoadjuvant regimens, including FOLFIRINOX, should be evaluated in randomized clinicals trials in this cohort of patients.

## Conclusions

Preoperative staging in terms of defining surgical resectability according to ICC is crucial for survival of patients diagnosed with PDAC. Apart from anatomical factors, prognosis of PDAC patients is substantially dependent on preoperative CA19-9 serum levels according to our data. Therefore, not only PDAC patients staged BR-A should be considered for neoadjuvant multimodal therapy but also BR-B patients, even if considered anatomically resectable. Future studies need to determine appropriate cutoff values for CA19-9.
